# Testing the effects of nutrition-related claims on perceptions of and preferences for alcohol products among Australian consumers

**DOI:** 10.1093/heapro/daag028

**Published:** 2026-03-02

**Authors:** Asad Yusoff, Alexandra Jones, Bella Sträuli, Paula O’Brien, Jacqueline A Bowden, Simone Pettigrew

**Affiliations:** The George Institute for Global Health, Faculty of Medicine & Health, University of New South Wales, Level 8, Health Translation Hub, 55 Botany Street, Randwick, Sydney, NSW 2031, Australia; The George Institute for Global Health, Faculty of Medicine & Health, University of New South Wales, Level 8, Health Translation Hub, 55 Botany Street, Randwick, Sydney, NSW 2031, Australia; The George Institute for Global Health, Faculty of Medicine & Health, University of New South Wales, Level 8, Health Translation Hub, 55 Botany Street, Randwick, Sydney, NSW 2031, Australia; School of Medicine and Dentistry, Griffith University, 1 Parklands Dr, Southport Gold Coast QLD 4215 Australia; Melbourne Law School, The University of Melbourne, 185 Pelham St, Carlton, Melbourne, VIC 3053 Australia; National Centre for Education and Training on Addiction (NCETA), Flinders Health and Medical Research Institute, GPO Box 2100 Adelaide, SA 5001, Australia; The George Institute for Global Health, Faculty of Medicine & Health, University of New South Wales, Level 8, Health Translation Hub, 55 Botany Street, Randwick, Sydney, NSW 2031, Australia

**Keywords:** alcohol, labelling, claims, policy

## Abstract

To appeal to health-conscious consumers, alcohol companies are marketing some products with a variety of on-pack claims. The aims of this study were to assess (i) whether the presence of sugar, carbohydrate, or energy claims on alcohol labels can influence perceived healthiness and product selection, (ii) whether any differences occurred by age and gender, and (iii) whether claim format influenced any observed effects. A total of 2034 Australian drinkers responded to an online survey where each participant was randomized to view products featuring either sugar (e.g. low sugar), carbohydrate (e.g. low carb), or energy claims (e.g. 86 cals). Participants viewed sets of three products from three different alcohol categories (out of a possible five: beer, cider, premix, spirits, wine) without any claims, and then viewed the same products, some with and some without claims from their assigned condition. For each set, participants selected a preferred product and rated all products on perceived healthiness. All products within each set contained identical alcohol content. The addition of a claim to product labels significantly increased mean perceived healthiness scores. The largest increase was observed for carbohydrate claims, followed by sugar and then energy claims. The presence of a claim did not uniformly influence product selection. The results of this experimental study indicate that displaying nutrient content and energy claims on alcohol labels has the potential to mislead consumers into perceiving such products as healthier options. Policymakers should restrict the use of claims to limit companies’ ability to market alcoholic beverages as healthier alternatives.

Contributions to Health Promotion

- This study provides unique insights into the effects of sugar, carbohydrate, and energy claims displayed on alcohol products.
- Respondents viewed products featuring claims as healthier than those without despite containing identical ethanol content.
- These results contribute to the very limited evidence base on alcohol claims, providing important insights for this emerging global policy issue.

## Introduction

In response to rising rates of health consciousness and decreasing rates of alcohol consumption across some demographic groups (Kraus *et al*. 2020, Holmes *et al*. 2022, Oldham *et al*. 2022), alcohol companies are attempting to market some of their products as healthier alternatives to traditional alcoholic beverages (Keric and Stafford 2019). One way companies are communicating this message is via on-pack labelling, including through the use of ‘better for you’ claims. These claims can be defined as on-pack text that provides information about any actual or purported health-related characteristics of the product (Brownbill *et al*. 2018). They may refer to various product features such as processing practices, ingredients, and nutritional qualities.

Two types of ‘better for you’ claims commonly used to highlight the nutritional qualities of alcoholic beverages are nutrient content (e.g. low sugar) and energy (e.g. low calorie) claims (Codex Alimentarius 1997, Keric and Stafford 2019). Recent research from Australia and Canada has found that approximately one-third of ready-to-drink alcoholic beverages (also known as premixes) displayed at least one nutrient content or energy claim (Demers-Potvin *et al*. 2023, Sträuli *et al*. 2023). In Australia, the context of the present study, the prevalence of sugar-related claims on new alcohol products entering the market has increased over the last decade (Haynes *et al*. 2024a).

The presence of claims on packaging is concerning because they may distract consumers from the harms associated with alcohol consumption, such as accidental injury, mental health disorders, and seven different types of cancer (Baan *et al*. 2007, Rehm and Imtiaz 2016). This effect has been found in experimental studies demonstrating that products with nutrient content and/or energy claims are perceived as healthier (Cao *et al*. 2023, Atkinson *et al*. 2024, Haynes *et al*. 2024b, Hobin *et al*. 2024) and lower in alcohol content (Cao *et al*. 2023) compared to identical products that do not feature claims. At present, there is limited research on whether these effects carry over into purchasing decisions for alcohol products (Hobin *et al*. 2024); however, evidence from food research suggests that claims can influence purchasing decisions and that consumers who are more health-conscious are more likely to purchase a food product when it features a nutrient content claim (Aschemann-Witzel and Hamm 2010, Bialkova *et al*. 2016, Oostenbach *et al*. 2019).

The effects of claims on product perceptions and consumption may be larger in groups who have greater health-related motivations, particularly younger people and women (Wardle *et al*. 2004, Callaghan *et al*. 2024). Public health groups are concerned about this issue as the widespread use of claims on product labels may act to halt the falling rates of alcohol consumption observed among younger age groups (Oldham *et al*. 2022) while encouraging consumption among women, who have historically consumed less than men (Keric and Stafford 2019, The Australian Bureau of Statistics 2023). Previous research in the Australian context appears to support these concerns, finding that younger consumers tend to perceive products featuring claims as healthier than those without claims (Haynes *et al*. 2024b, 2025a). Differences in the effects of claims by gender, however, are less clear and seem to vary by the type of claim. For example, it has been shown that relative to women, men hold greater misperceptions about the healthiness of products featuring vegan and gluten-free claims, while women appear to more frequently perceive products featuring sugar claims as healthier options (Haynes *et al*. 2024b). It is unclear why these differences exist; however, it has been suggested that products marketed as being low in sugar content may appeal to female drinkers due to societal pressures to maintain low body weight (Pitt *et al*. 2023). There is limited research investigating whether these differences persist in experimental settings; however, one study has shown that women are more likely than men to try alcohol products featuring claims (Forbes *et al*. 2025). Further quantitative research is needed to examine how the effects of claims vary by age and gender, and whether different types of claims (e.g. carbohydrate) influence the perceptions and choices of certain groups (e.g. women) more than others.

Claims on alcohol packaging typically appear in one of two formats: quantitative or qualitative (Liu *et al*. 2019). Quantitative claims specify an exact amount and unit of measurement (e.g. 0 g sugar), while qualitative claims offer a general description without a precise measure (e.g. low sugar). Although qualitative terms such as ‘low’ and ‘less’ are subject to strict definitions under labelling legislation in several countries (Wartella *et al*. 2010, Health Canada 2022, Bryant Research 2025, Food Standards Australia New Zealand 2025), consumers often misinterpret claims presented in this format (Patterson *et al*. 2012). Specifically, evidence from the food labelling literature indicates that consumers can overestimate the degree of nutritional improvement suggested by qualitative claims due to the inherently vague and ambiguous nature of the terminology used (Liu *et al*. 2019). At present, however, there is limited evidence to indicate whether these effects also occur in the case of alcohol labels.

Restrictions on nutrient content and energy claims on alcohol labels are an emerging area of regulation, with countries pursuing a variety of approaches. In the European Union (EU) and the UK, nutrient content claims on alcoholic beverages are prohibited while energy claims are permitted (Department of Health and Social Care 2024, EUR Lex, 2024). The restrictions on nutrient content claims are based on concerns that promoting alcohol for its nutritional properties may downplay the significant health risks associated with consumption (The European Union 2006, Department of Health and Social Care 2024). In contrast, the USA and Canada permit the use of nutrient content and energy claims (Canadian Food Inspection Agency 2022, TTB: Alcohol and Tobacco Tax and Trade Bureau 2024), while in Australia, such claims are permitted but only regarding energy, carbohydrate, sugar, and gluten content (Australian Government 2023).

The lack of an international alcohol labelling standard was recently raised as an issue at the Codex Alimentarius Commission (CODEX) at the 48th Session of the Committee on Food Labelling (Codex Alimentarius Commission 2024). The World Health Organization (WHO) representative reported to the Committee that a majority of countries had expressed support for a standard about restrictions on health and nutrition claims. However, the chairperson of the Committee recorded that there were mixed views among country delegations about whether new alcohol labelling standards were needed or whether the current CODEX standards for ‘food’ adequately covered alcohol. As of mid-2025, this issue appeared to be on hold. The global standardization of alcohol labelling requirements has the potential to enhance public health, if standards are grounded in robust evidence to ensure consumers are not misled and are provided with accurate on-pack information that allows them to make informed choices about alcohol (Department of Health and Social Care 2021, The European Union 2024a).

Previous research examining the impact of nutrient content claims on consumer perceptions and decision-making has largely focused on specific types of claims (e.g. sugar; Cao *et al*. 2023), particular demographic groups (Cao *et al*. 2023, Haynes *et al*. 2024b, 2025a), or certain types of alcohol (e.g. premix drinks; Cao *et al*. 2023, Hobin *et al*. 2024, Forbes *et al*. 2025). Further research is needed to determine whether these effects extend to the broader drinking population, a wider range of nutrient content claims, and different claim formats (i.e. quantitative vs. qualitative). To assist in addressing these gaps, the aims of this exploratory study were to investigate (i) whether sugar, carbohydrate, and energy claims on alcoholic beverages influence consumers’ perceptions of product healthiness and their product choices, (ii) whether any differences occurred by age and gender, and (iii) whether claim format influenced any observed effects.

## Methods

This study was not pre-registered but followed a previously established methodology for testing the effects of nutrition labelling on food products using a quasi-within-subject design (Pettigrew *et al*. 2023). Ethics approval was obtained from a University Human Research Ethics Committee and all respondents provided informed consent prior to participation.

### Participants

In total, 2034 individuals were recruited via an International Organization for Standardization (ISO) certified panel provider (Pureprofile) in October 2024. Ethical approval was provided by the University of New South Wales Human Research Ethics Committee. Eligibility criteria required respondents to reside in Australia, be aged 18 years or older, and consume alcohol at least once per month. Quotas were applied to ensure the sample reflected the age and gender distribution of the Australian adult population. Based upon previous work, it was calculated that a minimum sample size of 228 participants in each condition was required to detect small effects with a statistical power of 80% (Shemilt *et al*. 2017, Jongenelis *et al*. 2018, Cao *et al*. 2023). A larger sample (*n* = 678 in each condition) was recruited to allow for multiple sub-group comparisons. As indicated in Table 1, randomization of respondents to one of the three conditions was successful.

**Table 1:** Sample characteristics, overall and by claim condition.

Variable	Total sample (*N* = 2034)	Sugar condition (*n* = 678)	Carb condition (*n* = 678)	Energy condition (*n* = 678)
*Gender*				
Male	49	50	49	47
Female	51	50	51	52
Other	<0.5	<0.5	<0.5	<0.5
*Age group (years)*				
18–24	11	9	12	10
25–34	18	19	18	17
35–44	18	20	16	17
45–54	16	15	17	17
55–64	15	14	16	15
65–74	12	12	11	14
75+	10	9	11	9
*Socio-economic status (IRSD* ^a^ *)*				
Low (deciles 1–4)	28	29	29	26
Medium (deciles 5–8)	38	38	37	39
High (deciles 9–10)	34	34	34	35
*Drinking status*				
Under or meets low-risk guideline	42	40	42	42
Exceeds low-risk guideline	58	60	58	58

^a^Australian Bureau of Statistics Index of Relative Socio-economic Disadvantage (Australian Bureau of Statistics 2025).

### Study design

**Figure 1:**
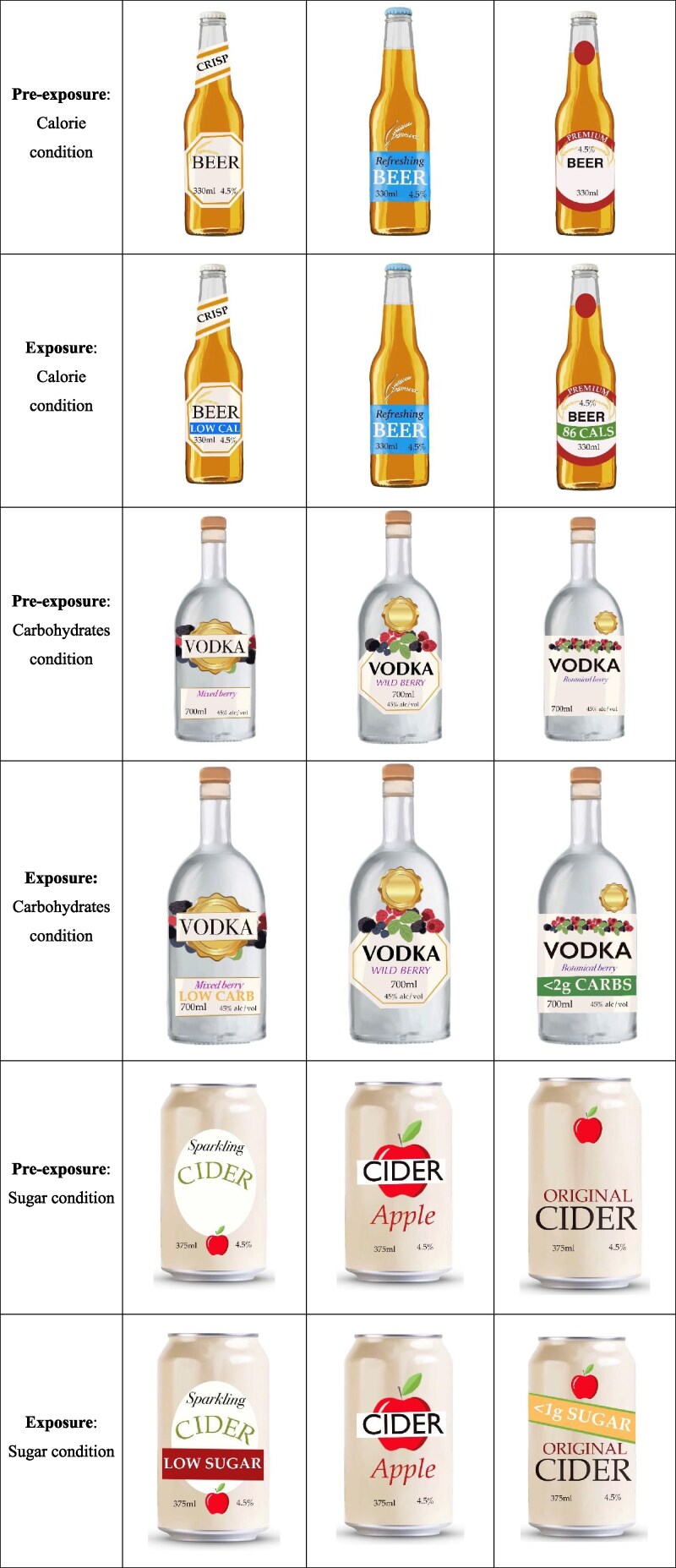
Examples of the stimuli used in the experimental survey.

Each survey respondent was randomly assigned to one of three claim conditions: sugar, carbohydrate, or energy. In the pre-exposure phase (see Fig. 1), respondents were randomly presented with three choice sets of three different alcohol types with no claims present on the mock product labels (e.g. 3 spirits products, then 3 cider products, then 3 beer products). All products within each set were identical in volume and contained the same amount of alcohol. The allocation of alcohol types to each claim condition was reflective of the current use of claims in the market (Haynes *et al*. 2024a). Respondents assigned to the sugar claim condition viewed wine, cider, and premix drinks; those exposed to carbohydrate claims viewed beer, spirits, and cider; and those who saw energy claims viewed beer, cider, and premix drinks. For each set of three products, respondents were asked, ‘Assuming you were interested in purchasing this type of drink, which of the following options would you most prefer to buy?’ (Pettigrew *et al*. 2023). They were then asked, ‘How healthy is this product?’, and asked to rate each product on a 5-point Likert scale ranging from ‘Very unhealthy’ to ‘Very healthy’ (Talati *et al*. 2017).

In the following exposure phase, this process was repeated with the same three sets of three products; however, two products in each set featured a nutrition-related claim relevant to their assigned condition (one quantitative claim and one qualitative claim), while the third product label remained unchanged (see Fig. 1).

### Data analysis

Data analysis was conducted using Stata18 (StataCorp 2023). To assess the influence of claims on product perceptions, mean perceived healthiness scores were calculated overall, by claim type, and claim format. To investigate whether perceived healthiness differed across claim formats, repeated-measures ANOVA was performed for each claim type. Post hoc pairwise comparisons with Bonferroni adjustments were conducted in cases where ANOVA testing was significant. To evaluate the effect of claims on product selection, the proportion of participants selecting each product was calculated by claim type and claim format.

To further investigate the effects of nutrition-related claims on perceived healthiness and any potential variations by age and gender, logistic regression analyses were conducted. (Note: due to limited variation in product selection outcomes, analyses were restricted to perceived healthiness). In line with previous research using logistic regression to analyse factors influencing perceived healthiness of unhealthy beverages (Drolet-Labelle *et al*. 2025), the dependent variable was dichotomized, with respondents who rated products as ‘healthy’ or ‘very healthy’ classified as perceiving them as healthy, while all other responses were categorized as not healthy (‘very unhealthy’, ‘unhealthy’, and ‘neither healthy nor unhealthy’). The neutral response was classed as ‘not healthy’ to allow the ‘healthy’ response category to only include participants who, contrary to the current WHO guidelines (World Health Organization 2024b), perceived alcohol as a healthy product. Respondents were categorized into three age groups: 18–34, 35–54, and 55+ years, with gender treated as a binary variable. Separate regression analyses were estimated for each claim type: sugar, carbohydrate, and energy.

To account for potential confounding variables, the regression models included covariates for socio-economic status, adherence to the Australian drinking guideline, time (i.e. pre-exposure vs. exposure to nutrition-related claims), and unobserved individual differences. Socio-economic status was measured using the SEIFA Index of Relative Socio-economic Disadvantage (Australian Bureau of Statistics 2025), with respondents classified into low, medium, or high categories. Guideline compliance was assessed against the Australian National Health and Medical Research Council’s threshold of no more than 10 standard drinks per week and no more than four standard drinks on any day (NHMRC 2025). To control for unobserved individual-specific characteristics—given that respondents served as their own controls—individual fixed effects were applied in the regression models.

## Results

### Perceived product healthiness

Mean perceived healthiness scores increased for all products once a nutrition-related claim was added to the label (Table 2). Averaging across both claim formats (qualitative and quantitative), the largest increase in perceived healthiness was observed in the carbohydrate condition (0.25 units on the 1–5 scale), followed by the sugar condition (0.24), then the energy condition (0.21). The proportion of respondents rating products as healthy increased from 13% to 26% for carbohydrate claims, 18% to 31% for sugar claims, and 21% to 33% for energy claims.

**Table 2:** Perceived product healthiness by claim condition and claim format.

% rating product as healthy*	Carbohydrate (*n* = 678)			Energy (*n* = 678)			Sugar (*n* = 678)		
	Pre	Post	Change	Pre	Post	Change	Pre	Post	Change
Control	15	13	−2	21	18	−3	18	14	−4
Qualitative claim	13	26	13	20	34	14	17	31	14
Quantitative claim	14	26	12	21	31	10	18	31	13
Average of both claim formats	13	26	13	21	33	11	18	31	13

Mean (SD)	Pre	Post	Δ	Pre	Post	Δ	Pre	Post	Δ
Control	2.70 (0.72)	2.69 (0.73)	−0.01^a^	2.91 (0.74)	2.87 (0.77)	−0.04^a^	2.87 (0.72)	2.79 (0.72)	−0.08^a^
Qualitative claim	2.68 (0.71)	2.95 (0.80)	0.27^b^	2.90 (0.73)	3.13 (0.82)	0.23^b^	2.84 (0.70)	3.09 (0.82)	0.25^b^
Quantitative claim	2.69 (0.71)	2.93 (0.82)	0.24^b^	2.91 (0.75)	3.09 (0.81)	0.18^c^	2.87 (0.72)	3.10 (0.83)	0.23^b^
Average of both claim formats	2.69 (0.69)	2.94 (0.79)	0.25	2.90 (0.72)	3.11 (0.80)	0.21	2.86 (0.70)	3.10 (0.79)	0.24

Superscript letters denote proportions within each column that significantly different from one another.

^*^Rated product as ‘healthy’ or ‘very healthy’ on a 5-point scale from 1 = ‘very unhealthy’ to 5 = ‘very healthy’.

Across all three claim types, the increase in mean perceived healthiness was greater for claims presented in a qualitative format than a quantitative format, although the difference was only statistically significant for energy claims. For all claim types, both qualitative and quantitative formats produced significantly greater increases in perceived healthiness compared to the no-claim products in the exposure phase.

These effects were similarly identified in the regression analyses that controlled for age, gender, socio-economic status, and drinking level. In all three regression analyses, the presence of a claim (averaged across qualitative and quantitative formats) led to a statistically significant increase in the likelihood of respondents rating products as healthy (see Table 3). The largest effect was observed for carbohydrate claims, where respondents were approximately three times more likely (OR = 2.77, 95% CI = 2.04–3.77) to rate a product with a claim as healthy compared to the same product without a claim. A similar effect was found for sugar claims (OR = 2.65, 95% CI = 1.92–3.65), with a smaller effect for products displaying energy claims (OR = 1.65, 95% CI = 1.30–2.09).

**Table 3:** Logistic regression results for perceived healthiness: odds ratios.

	Carbohydrate (*N* = 12 168)		
	Odds ratio	95% CI	*P*-value
Claim	2.77***	2.04–3.77	0.000
Female	1.25	0.96–1.61	0.095
Age			
18–34	0.81	0.57–1.14	0.222
35–54	0.63**	0.45–0.88	0.007
55+	REF	NA	NA

	Energy (*N* = 12 150)		
	Odds ratio	95% CI	*P*-value
Claim	1.65***	1.30–2.09	0.000
Female	1.39**	1.10–1.75	0.006
Age			
18–34	1.23	0.91–1.67	0.171
35–54	1.09	0.82–1.46	0.558
55+	REF	NA	NA

	Sugar (*N* = 12 168)		
	Odds ratio	95% CI	*P*-value
Claim	2.65***	1.92–3.65	0.000
Female	1.27	0.98–1.65	0.065
Age			
18–34	1.08	0.76–1.51	0.675
35–54	0.85	0.61–1.19	0.348
55+	REF	NA	NA

*N* = number of product-level observations for each regression analysis. Each participant had multiple observations across multiple alcohol types. In cases where coefficients included in the model were insignificant across all three conditions/regressions, figures are not reported. Due to insufficient sample size, individuals who did not identify as ‘Male’ or ‘Female’ were not included in the regression analysis.

**P < 0.01, ***P < 0.0001.

Across all three claim types, age and gender were not consistently associated with respondents’ responses, and effects were only statistically significant in two instances. First, in the energy claim condition, female respondents were 1.39 times more likely than males to rate a product as healthy when exposed to a claim. Second, in the carbohydrate claim condition, respondents aged 35–54 years were 1.59 times less likely to rate a product with a claim as healthy compared to those aged 55 years and older.

### Product selection

As shown in Table 4, only the presence of a sugar claim resulted in significant changes in product selection, but in opposite directions for the qualitative and quantitative claim types. The qualitative sugar claim resulted in a 6 percentage point increase in choice prevalence compared to the pre-exposure condition, while the quantitative sugar claim produced a −7 percentage point decrease.

**Table 4:** Product selection by claim type and claim format (%).

	Carbohydrate (*n* = 678)			Energy (*n* = 678)			Sugar (*n* = 678)		
	Pre	Post	Δ	Pre	Post	Δ	Pre	Post	Δ
Control	44	44	0	36	36	0	35	36	1
Qualitative claim	27	27	0	28	29	1	28	34	6
Quantitative claim	30	29	−1	36	35	−1	37	30	−7

## Discussion

Consistent with previous research, our study found that the presence of nutrient content and energy claims on alcohol labels has the potential to mislead consumers about the healthiness of alcoholic beverages (Cao *et al*. 2023, Hobin *et al*. 2024, Haynes *et al*. 2025b). Products featuring such claims received higher healthiness ratings than products without these claims, despite all products having the same alcohol content. These results add to the literature by isolating the effects of sugar, carbohydrate, and energy claims, with carbohydrate claims having the largest impact on perceptions of an alcohol product’s healthiness, followed by sugar and then energy claims. Our results suggest that across all three claim types, consumers may be unable to recognize that products with identical ethanol content are equally unhealthy in terms of the risks associated with alcohol consumption. While this may be due to consumers’ lack of awareness of many of the harms associated with alcohol consumption (McNally *et al*. 2019, Wiseman and Klein 2019, Kokole *et al*. 2023), it may also occur by on-pack claims diverting consumers’ attention away from critical health-related information, such as the alcohol content, and instead drawing consumer attention to other product features (Cao *et al*. 2023, Pitt *et al*. 2023, Haynes *et al*. 2024b, 2025b, Hobin *et al*. 2024). In both cases, however, rather than promoting informed decision-making, the use of claims may undermine consumers’ ability to accurately assess the potential harms associated with alcohol products (Cao *et al*. 2023, Hobin *et al*. 2024).

A key principle of the existing general Codex labelling guidelines for pre-packaged food (including alcohol) is that ‘products should not be described or presented on any label or in any labelling in a manner that is false, misleading or deceptive’ (Codex Alimentarius Commission 1991). Our study suggests that these general Codex labelling guidelines—originally designed for packaged foods—are inadequate for guiding the regulation of claims on alcoholic beverages. To effectively protect consumers, it is essential to develop alcohol labelling standards (both at the international level through Codex and at the country level) that (i) restrict the use of nutrient content, energy, and other potentially misleading claims on alcohol labels and (ii) regulate which and how information can be given to consumers.

A novel contribution of this study is the examination of how claim format (qualitative vs. quantitative) influences consumer perception. Evidence from the food labelling literature suggests that consumers can overestimate nutrient reductions implied by qualitative claims, indicating a poor understanding of the meaning of terms such as ‘low’ and ‘lower’ (Liu *et al*. 2019). Our findings partially support this notion, as products with a qualitative energy claim were perceived as healthier than those with a quantitative claim. Future research should examine how claim format influences consumer perceptions and decision-making in realistic shopping environments where individuals are typically exposed to more information than they can process (Buratto and Lotti 2023), and therefore may preferentially look for on-pack information presented in a simpler format (van Kleef *et al*. 2008).

While the marketing of products with nutrition-related claims appears to be often targeted at younger and health-conscious drinkers (Hanrahan-Lawrence 2024, Euromonitor International 2025), we found that responses to the claims were relatively consistent across the sample. With a growing number of consumers from a variety of demographic groups looking to moderate their alcohol consumption (International Wine and Spirits Record 2024), it is critical that claims about the nutritional qualities of products are not used to distract consumer attention from information about alcohol content. Even at low levels of consumption, alcohol is associated with significant increases in the risk of developing oesophageal, colorectal, and breast cancers (Baan *et al*. 2007)—risks that remain largely unknown to many who drink (Hopkins 2023, Kokole *et al*. 2023). The heavy use of claims by the industry and subsequent effects on perceived healthiness can be likened to previous efforts by the tobacco industry to promote ‘light’ and ‘mild’ cigarettes as safer options (Australian Competition and Consumer Commission 2005). As a result, it is essential that policymakers take steps to restrict the use of claims—not only to protect at-risk groups such as younger drinkers, but also the broader drinking population.

At present, there is conflicting evidence as to whether on-pack claims influence intended or actual product consumption (Forbes *et al*. 2025, Haynes *et al*. 2025b). However, the findings of the present study are consistent with previous work among young Australians, suggesting that the inclusion of claims on alcohol products does not significantly influence product selection (Haynes *et al*. 2025b). Despite these findings, this does not diminish the concern that such claims have the potential to mislead consumers about the healthiness of alcohol, which as aforementioned would be inconsistent with both Codex guidelines and principles outlined in general consumer law across several nations (Codex Alimentarius Commission 1991, Australian Competition and Consumer Commission 2023, The European Union 2024b). The potential for these claims to shape consumer perceptions about alcohol and influence consumption choices remains problematic and raises concerns regarding the types of information provided to consumers on product labels.

The primary limitation of this study was its experimental nature, which may restrict the generalizability of the findings to real-world contexts. Second, due to the nature of the questionnaire, respondents could have guessed its purpose, potentially biasing their response towards rating products with claims as healthier than they actually believed them to be. Third, participants were not asked what type of alcohol they regularly consumed, which may have influenced responses given the product randomization process within the survey meant they did not necessarily view types of alcohol they consumed regularly. Finally, only a small subset of claims commonly used by alcohol companies was tested. Future research should investigate the impacts of other commonly featured claims on alcohol products (e.g. preservative claims), assess whether the presence of multiple claims amplifies the effects found in this study, and examine whether on-pack claims reduce the impact of on-pack warning labels.

In conclusion, the results of this study show that the use of nutrient content and energy claims on alcohol labels has the potential to mislead consumers about the healthiness of alcoholic beverages. The widespread use of such claims in the alcohol market may undermine recent reductions in alcohol consumption, particularly among at-risk groups such as younger drinkers. To prevent certain alcohol products from being promoted and perceived as healthier options, policymakers should consider restricting the use of these claims on alcohol packaging and in related marketing materials.

## Authors’ contributions

Conceptualization: A.Y. and S.P.; Methodology: A.Y., S.P., and B.S.; Formal analysis and investigation: A.Y.; Writing—original draft preparation: A.Y.; Writing—review and editing: A.Y., S.P., B.S., A.J., P.O.B., and J.B.; Funding acquisition: S.P., P.O.B., A.J., and J.B.

## Data Availability

Data access will be provided on reasonable request.
